# Transactions of the Obstetrical Society of Cincinnati

**Published:** 1880-05

**Authors:** 


### Reports of Societies.

### TRANSACTIONS OF THE OBSTETRICAL SOCIETY OF CINCINNATI.

Stated meeting, March 11th, 1880. The President, Thad. A. Reamy, M.D., in the chair.

Dr. Illowy read a paper on the etiology of puerperal eclampsia, after which the following remarks ensued:

Dr. Underhill—There is more said concerning puerperal eclampsia, in proportion to the limited knowledge we have of that affection, than of any other disease pertaining to the pregnant or puerperal state. There is but little better agreement about its treatment than in regard to its etiology. Medical authorities do not even agree as to what constitutes the disease. Most of them speak of different classes of convulsions, as, for example, the apoplectic, the hysterical, the tetanic, and the epileptiform. Others, Playfair prominently among the number, exclude the first two from the category, and regard them not as true eclampsia, although they admit that "the paroxysms, while they last, are the same essentially as those of an epileptic fit." Playfair states that the convulsions of the hysterical type "are identical" with these forms of convulsions in the non-pregnant state, "and are in no way special to their nature."

To me it seems that these two classes are as truly eclamptic as is the epileptiform or tetanic class, since they depend, so far as is known, upon the same or similar remote causes, when associated with the puerperal or pregnant state? As well might we rule out such diseases as puerperal septicemia and pyemia, and relegate them to the field of surgery as septicemia or pyemia of purely traumatic origin. Other maladies might be named which belong particularly among the puerperal diseases, yet sometimes occurring in the non-pregnant condition, are not on account of the latter fact banished from the category of puerperal affections.

From what has been said in regard to the propriety of considering each of the different classes of convulsions belonging to pregnancy and parturient state as truly eclamptic, it follows that there often arises difficulty of diagnosis. Some do not belong distinctly to the hysterical type, yet they more nearly resemble that form than either of the others. Again, a series of convulsions may approach the epileptiform in their symptoms, and yet be not clearly of that class. Similar statements are true concerning the other two varieties. They are not always distinct and clearly defined, and the different types graduate into each other; or the convulsions, in a given case, may partake of the nature of either two of the different forms named. Puerperal eclampsia, in which the spasms simulate those of epilepsy, cannot always be distinguished at first sight from epileptic fits. Even if it be learned that the patient is an epileptic, it does not necessarily follow that it is a case of eclampsia wherein the convulsions take the epileptiform character, as it does probably in consequence of the old epileptic habit. When death results in a case of this kind, autopsy shows lesions in the kidneys and in the brain, of the same character, and as constantly as they are found after death from true eclampsia in those cases wherein no previous epilepsy had existed.

In this connection I may state that I am personally cognizant of two cases of epilepsy of long standing, in which, during several pregnancies, no epileptic seizures took place in either of the women. In these individual examples pregnancy seemed to interrupt completely the fits, which, however, always again returned after recovery from parturition. I am not aware of similar cases having been noted by others, nor do I attempt to explain the fact witnessed in these instances. It is certain, however, that epilepsy does not uniformly cease during the pregnant condition, as those of us know who have had much experience in treating this disease. One case I recollect especially to which I was called at about the seventh month of gestation. She had suffered from epileptic attacks since childhood, but they grew so frequent and violent duriug pregnancy that medical aid was sought. She, however, died in one of the seizures before the completion of her term. There was no threatening of premature labor, no albumen in urine, nor other evidence of true eclampsia either of the epileptiform or other variety.

Other gentlemen of this Society have discussed the pathogenesis of puerperal convulsions with greater ability than I can bring to this branch of the subject. Yet I may add that dystocia, so often claimed to be a causative agent, has, in the opinion of those best entitled to know, little or no influence in the causation of the convulsive disorder. Usually where eclampsia occurs the labor is normal.

1. Convulsions take place three times as frequently among primipara as among multipara.

2. Primipara of advanced years are more liable to attack than those of earlier age.

3. Mental distress favors its occurrence.

4. Multiple pregnancy is much more likely to be attended with convulsions than single gestation. Statistics show that twin pregnancies are seven times as frequent with the eclamptic as with the non-eclamptic.

Of the theories advanced to account for the phenomena attending true eclampsia, I believe neither of them will axplain *all* cases. A small proportion admit of explanation by the so-called uremic theory of Frerichs, which has been so warmly advocated by Hecker and Litzman. A far larger number are explained satisfactorily by the view so ably promulgated by Traube, Rosenstein, and Munk, who attribute the phenomena to the condition of the brain, so well set forth by those members who in this discussion have referred to that theory. Another explanation, that of the late Dr. Tyler Smith, as quoted and endorsed by Playfair, claims that convulsions are remotely "caused by the peculiar excitable condition of the nervous system in pregnancy." But as to the essential nature of this peculiar excitability we are left in the dark. Nor is this "peculiar excitable condition" always manifested, at least so far as the patient or her associates are able to discover. High authority, as well as some of my *confreres*, have given their adherence to this view, which to me, I must confess, seems to explain nothing, being merely a form of words to cover our ignorance.

Briefly, then, I believe that all cases of puerperal eclampsia are due to affections of the brain or kidney—far more frequently the former. In this particular I am more of a humoralist than a solidist.

Of course it is admitted, that in nearly all cases albuminuria exists, yet the renal lesion may not be sufficient to excite the convulsions which recur. Besides, as has been pointed out by others during this discussion, it is well known that eclampsia may occur without albuminuria.

When cerebral hemorrhage takes place in a case of puerperal convulsions, as in one of Dr. Reamy's, it is an interesting point to determine whether the hemorrhage is secondary to the convulsion, or vice versa. Usually the clot is small, often only the size of a pea ; but Liddell, in his treatise on apoplexy, relates a case of puerperal eclampsia in which, upon post mortem, was found a clot weighing two ounces.

The cerebral blood-vessels are subjected to great tension by the hypertrophy of the heart known to exist in pregnancy and by the strong muscular movements occurring during the pains of labor. Add to this tension the increased arterial pressure caused by the tonic spasms of the whole muscular system in eclampsia, and we have force enough to rupture the cerebral vessels. Hence, it seems fair to conclude that the apoplexy is, in much the larger number of instances, secondary to the convulsion.

I need not refer to the symptoms of this convulsive disorder but I desire to say something concerning its treatment. It may be treated by

(*a*) Venesection.

(*b*) Sedatives and narcotics.

(*c*) Enemata and drastic cathartics.

(*d*) Diuretics.

(*e*) Diaphoretics.

Concerning venesection, I believe the medical pendulum has vibrated to a point opposite the extreme limit it had reached many years ago. I favor restoring Dr. Gross' "Lost Art' in the treatment of this affection. Because the remedy has been abused is no reason why it should be altogether abandoned. A plethoric woman will be benefitted usually by general blood-letting, especially if resorted to early. It is seldom that the bleeding should be repeated, and it is rarely or never required after delivery.

I have changed my views somewhat in reference to general blood-letting in these cases. Those that I have treated were not bled from the arm, partly because I did not then think this remedy appropriate to the treatment of eclampsia.

It is evident to all that a prominent indication is to relieve the increased pressure on the arterial system. This pressure is the *immediate* cause of the convulsion, no matter what may be its remote origin. Venesection relieves that pressure, temporarily at least.

Arterial sedatives, such as *veratrum viride*, of course, diminish the frequency and power of the heart's action, thereby relieving, to a certain degree, the pressure in the cerebral blood-vessels. The *bromides of potassium*, sodium and ammonium do good, not only by lessening the calibre of the vessels in the brain, thereby decreasing the amount of blood circulating there, but they also oppose reflex excitability. *Chloroform* controls the violent muscular actions which so greatly tend to increase the hyperæmia, and is of great value during the paroxysm. *Chloral* is a standard remedy, but I have little faith in its efficacy. It will be seen by reference to case I., reported below, that I gave it persistently, but without appreciable benefit. The theory, so ingeniously propounded by Leibreich, and supported by Richardson, that chloral was slowly converted into chloroform by the alkali in the blood has long since been exploded. Stille has just pointed out many and conclusive reasons, which I need not here name, why chloral is not converted into chloroform in the circulation.

When inhalation of chloroform fails to relieve convulsions, *hypodermic injections of morphia* are often of great service. The remedy should be given in large doses, repeated with sufficient frequency to render the narcotic effects continuous, thereby better controlling muscular action than if used tentatively.

The other measures to be used for the purpose of lessening the blood-pressure on the brain, such as enemas, (which act also as revulsives) croton oil, elaterium and other drastic purgatives, agents to promote excretion by the kidneys are important, and also agents to supplement the action of these organs by securing the diaphoretic action of the skin• But these are subsidiary to the first three classes of remedies named. Besides the summary of treatment just sketched, if the brain be greatly congested, ice poultices or vesicants, on the nape of the neck, cold compresses or the cold douche on the head, may be advisable. Local bleeding behind the ears or on the forehead may tend to diminish the danger.

Finally, there arises the important question : Shall we empty the uterus ? This interrogatory has been answered by eminent authorities both in the affirmative and negative. Of course, it is in the interest of the child that delivery be accomplished as speedily as possible. And it seems to me that the contents of the uterus must prove a source of irritation to the mother and should, therefore, be removed as early as practicable with safety to her. But we must see to it that in our efforts to complete labor we do not cause a greater irritation than that which we seek to remove.

Gentlemen, I beg to supplement my remarks with reports of two cases of which I fortunately have preserved notes. They are of very different types—the first partaking of the character of both the hysterical and epileptiform varieties, and the second of the "apoplectiform."

Case I.—On the morning of June 20th, 1875, was called to see Mrs. T. who, as was stated by the messenger, was at that time in a convulsion. At my visit, which was the first time I had ever attended the lady, I found she was just returning to consciousness. Knowing nothing of her or her family, I at first suspected it to be a hysterical spasm, especially as she was of nervo-sanguine temperament and suggested at first sight the general aspect of the hysterical woman. She was 20 years of age, a teacher in our public schools, and had been married about six months. Her duties had been very arduous. She had a room in which the children were difficult to be governed; and, as the school year was about to close, she had strained all her energies to bring her class up to the percentage necessary for passing to the next grade.

Further inquiry elicited a knowledge of the fact that she was five months *enciente.* Before leaving patient I was satisfied it was a case of puerperal eclampsia, although I had not anticipated finding convulsions at so early a period of pregnancy. Ordered large doses of calomel and jalap, to be assisted in its action by an anema. Also chloral in 15-grain doses, combined with scruple doses of potass. bromide every two hours till evening visit.

During the day she had several convulsions, and one during my visit in the evening. It assumed somewhat the tetanic form, combined with the hysterical variety. Later, the convulsions appeared to be more of the epileptiform and hysterical kind.

General blood-letting did not seem to be called for, the patient appearing the reverse of plethoric. Still, there were such evident marks of cerebral congestion that I ordered free leeching to the temporal and mastoid regions. This was supplemented by wet cups to nape of neck which answered a two-fold purpose of depletion and counter-irritation. Chloral and bromide of potash was continued, but at longer intervals.

*June 21st.*—One o'clock A. M. Was called by Mr. T., who assured me his wife was dying. On repairing to his house I thought he was correct in his opinion. One convulsion rapidly succeeding another, and each, it seemed at the time, must be fatal. Found the os partially dilated and yielding. Manual dilatation soon made it possible to use the forceps, and in half an hour from entering the house I delivered her of a living babe, which, however, soon, died in consequence of its immaturity. Just after delivery she experienced the last and most frightful convulsion of the whole series, which was eighteen in number. Recovery gradually took place without the occurrence of any serious complications.

In this case many more visits were made than I have indicated in its report. Other remedies than those already named were also used, such as diuretics, arterial sedatives, and diaphoretics. Neither chloral, the bromides, cold affusions, ice bags, nor any other agent employed, seemed to have much effect. Convulsions ceased only after the expulsion of the placenta; and hastened delivery, in my judgment, alone saved life.

The pupils, which in the beginning were contracted, dilated to the natural size after local abstraction of blood.

The urine contained 80 per cent. albumen. There was no dropsy of the subcutaneous cellular tissue when I first saw the patient, but puffiness of the face and upper extremities soon thereafter made its appearance. There was much nausea and vomiting; temperature was not found above 103°. A typhoid condition ensued, lasting a week or ten days, during which, after return to consciousness, she complained greatly of headache.

Although nearly five years have elapsed since the attack, Mrs. T. has never yet been able to recall any incident that transpired from the date of the inception of her illness till the next day after delivery. Eighteen months subsequently I attended her in confinement again, and there were no indications of the slightest tendency to eclampsia.

Case II.—October 25th, 1876. At half-past one o'clock A.M., called to attend Mrs. L. in confinement. Knew nothing of the patient previously, but found her a multipara, æt. 30. She did not think it time by a fortnight for accouchement, and attributed—perhaps rightly—the onset of labor to hypercatharsis which had been induced by a drastic purgative taken the evening previously. Excessive nausea and slight vomiting she attributed also to the same causes, which developed the eclampsia.

Examination showed labor to be in first stage; vertex L. O. A.; os rigid—dilated only to size of silver dollar; membranes had ruptured spontaneously prior to my arrival; waited three-quarters of an hour and examination now showed but little relaxation of the os; gave a scruple dose of chloral and returned home, leaving instructions that I should be summoned whenever the attendants might deem it necessary. I thought labor would terminate about six A.M.

Mr. L. came for me a few minutes before five A.M.; and upon going to his house I found his wife in tonic spasms. The women in attendance informed me that at 4½ o'clock suddenly all labor pains apparently ceased. In a few minutes thereafter she cried in great agony, "Oh, my God! my head is opening." Then she complained of a choking feeling; next her speech became thick, and articulation in a few minutes impossible. Just before her complaint of the agonizing pain in the head she rubbed her face and complained of its itching in a very annoying way. Almost as soon as articulation ceased she became unconscious. These are the statements made to me by those who were with her, and all the symptoms just described occurred within a period of twenty minutes. I found her breathing sterterously, Left pupil very much dilated; right normal; hands tightly clenched; and carpopadel contractions existed to a marked degree. Every muscle of the body was so rigid as to be as hard as a board. Now, for the first time, I noticed the condition of the feet and legs, and found that they were much swollen. By inquiry I learned that they had been greatly swollen for the last three weeks. With catheter I obtained enough urine to test, which, upon boiling, almost solidified. I gave chloroform by inhalation, though very cautiously, as the weak, slow pulse, pale face, and general appearance contra-indicated the free use of an anæsthetic. Deglutition was impossible in her comatose condition. At 5½ o'clock (A.M.) she had the only pronounced convulsion which I witnessed, and that was of the apoplectiform type. The head was drawn down on the right shoulder, and the most frightful muscular contortions took place. After this the breathing was even more stertorous if possible than before. Auscultation for fetal heart showed that the child was probably dead; but as there was now sufficient dilatation to apply the forceps I resorted to them, and delivered the dead fetus, thinking that if this source of irritation was removed it would be better for my patient.

The muscular system remained in tonic spasm till after 6 o'clock. when the general rigidity began to yield. The convulsive disorder relaxed; she gradually sank, remaining in a profoundly comotose state until nine o'clock, when she died. Death took place ten hours subsequent to beginning of labor, seven and a-half hours after I first saw her, and three hours after delivery.

Dr. Tate :—The subject which the Society has under consideration is one of great importance, and as such must attract the attention of every man engaged in obstetrical practice. It is a disease that is the more alarming, because of the suddenness of its eruption in the midst of apparent health, and because of the horrid symptoms by which it is attended. I have seen more than twenty cases of these convulsions, but have never had an opportunity of witnessing a post-mortem examination of but two of them. In one of these, which had been treated by a physician without venesection, the woman died apparently from intense cerebro spinal congestion and an effusion of blood along the medulla oblongata and spinalis. In the other case the patient had been freely bled, and died in a state of coma without sanguineous effusion. In this case the kidney was especially interrogated by one of our special pathologists, and was found in a high state of congestion, *without any trace of inflammation.* In both cases the urine was loaded with albumen. The relation which this disease has to albuminuria (first pointed by Lever in 1843) has impressed itself very deeply upon my mind, and consequently I have instituted in my service, in the City Hospital, an investigation into the conditions of the urine of pregnant women. In 82 cases the urine of these women has been examined. The specific gravity ascertained, and the fact exhibited as to whether it contained albumen or not. It is to be remarked that (in consequence of moral circumstances not necessary to mention) seventy-five per cent. of women delivered there are primipara. The specific gravity of the urine was found to vary from 2—24, the great majority of cases being about thirteen, and the urine almost invariably acid. In these 82 cases the urine contained a trace of albumen in ten cases, a considerable quantity in one case, and a large portion in one. Thus we have 14 per cent. for all cases, and only 2½ per cent. for those where there was any considerable quantity of albumen.

These investigations were made mostly by the internes of the hospital, who tested the urine by heat and nitric acid; but thirty of the cases were re-examined by myself, and thirteen of them by Mr. Fennel, one of our best analytical chemists. In these latter cases examined, both by Mr. Fennel and myself, there were ten consecutive examples of the urine of primipara without a trace of albumen in that fluid. In the only case where there was a large amount of albumen the patient died, in five hours after admission into the hospital, of puerperal eclampsia.

I consider that these results are the most important because they differ so widely from the experiment of Mons. Blot, who says he found albumen at the rate of thirty per cent. in the urine of the primipara.

Now, you must have observed in reading the works of Bedford and Leishman, and in most of the papers on eclampsia, recently published in our medical journals, that the opinions of Blot and Litzman, are taken as authority on this subject, and that they have completely dominated over the professional mind, so that now it seems to be generally thought that the presence of albumen in the urine (especially in the primipara) is of such ordinary occurrence, that it is to be regarded as a thing of no special importance. On the contrary its presence at any time should, I think, excite grave apprehension, and, if it appear in any considerable quantity (especially if attended by a diminuition in the amount of urine) it should fill the mind of the accoucher with the gravest forebodings.

Upon the general subject of puerperal eclampsia I would say that the progress of physiology has shown that the disease is located in the true spinal cord—that is, in the medulla spinalis, oblongata, pons, crura cerebri and tubercula quadrigemini.

The morbid state of these nervous centers seems to consist in a high degree of impressibility and excitability, accompanied by congestion, which conditions have been induced by the circulation of blood, which has not been depurated of effete materials, *in consequence of a failure in the functions of the kidney.*

The condition of the nervous centres I look on as the great and only predisposing cause leading to puerperal convulsions. Whenever this is found the cord is liable at any time to be thrown into convulsive paroxysms by a dozen different exciting causes. We may have puerperal irritation from the distension of the uterus, from incipient contractions from pressure on the perineum, from clots in the womb, from undigested materials in the stomach, from distension of the bladder, or from a strong depressing moral emotion ; the brain itself acting as a peripheral nerve and sending down the shock on the centres of motility. This, in my judgment, is about all the brain proper has to do with inducing any case of puerperal eclampsia.

It appears, too, from the history of these cases, that in most of them there is no structural disease of the kidney whatever ; for we find in many of them, that in a few hours, or at most days, the urine will resume its normal character, showing that the kidney could only have been in a high state of congestion.

This disease occurs chiefly in primipara ; that is in persons in the full enjoyment of health and activity, most of whom in fact never felt better or looked so well in their lives. The blood vessels are full to repletion—though the serum is relatively greater in proportion to the solids than at other periods of life. Looking at the conditions of the persons of those who are seized with this malady, I am inclined to the opinion that in most instances the disturbance of the kidney is effected by backward pressure, either on that organ or its emulgent veins. The few cases that occur in early gestation are either those where there is a permanent structural affection of the organ, or where some reflex irritation from a peripheral nerve, as of the womb or ovarium, has brought on acute congestion or the kidney. We see this often occur to the stomach and bowels, and do not know why it may not happen in the kidney also.

In regard to the treatment of this affection we would say that it seems to us the indications are clearly to relieve the congestion of the kidney and nervous centres, and to prevent serous or sanguineous effusion into the parts of the brain within the cranium. The danger of this last is mostly occasioned by the constriction of the vessels returning the blood from the head, by reason of the contraction of the muscles of the neck. In all cases occurring before delivery the lancet should be at once employed, as the most rapid means of reducing the volume of the blood, and thus relieving internal congestion. After this, chloroform or ether should be brought into requisition in order to cut off any peripheral irritation. Croton oil should be given to produce purging and derivation from the head. To allay irritation the bromides and chloral may be used. If still the convulsions continue, the membranes should be broken, the os dilated and the womb emptied. In cases coming on after labor, it will seldom be necessary to bleed. Purging, however, will be useful, anæsthetics may be employed together with the bromides, and we should be especially careful to hunt out all sources of peripheral irritation, and to remove them. In cases where there is profound coma, I should be inclined to resort to hypodermic injection of pilocarpine in order to carry off the serous effusions, and even to remove some of the solid constituents of the blood. We know that in some cases of urinary suppression the smell of urine can readily be detected in the sweat.

